# The Chevalier de Butel on Plague in the Eastern Countries

**Published:** 1826-07

**Authors:** 


### THE CHEVALIER DE BUTEL ON PLAGUE.*

Although we have no hope that the ultra-declaimers against contagion, quarantine, &c. will ever listen to any facts which militate against their own doctrines, still we deem it proper, from time to time, to oppose facts to arguments, lest, by mere dint of repeated assertion, the latter should usurp a place in men's minds to which they are not entitled by their own intrinsic merits. Our Gallic brethren, who were long good Catholics in the creed of pestilential contagion, as well as in certain other creeds, have at length been *infected* with the doctrines of Maclean, now when they have entirely lost all reputation in the minds of the profession on this side of the channel—just as Brunonianism began to flourish abroad after it had fallen into decay at home. The contagionists and anti-contagionists are ranged in hostile array throughout the medical societies of Paris, as they were for a while in this country—where they now only occupy the columns of newspapers, having been driven from the sober discussions of medical men.

The paper which we are about to notice, is from the pen of the Chevalier de Butel, who resided fifteen years at Alexandria, and nine at Constantinople, where he had ample means of making most minute observations on the plague of the east. We shall endeavour to condense the more important facts into as small a space as possible. After observing that the universal opinion in Egypt is, that the plague is imported there from Constantinople; he states that he arrived at Alexan-

* Le Chevalier de Butel. Journ. Univers. Jan. 1826.

dria in the year 1787, at which period Egypt had been entirely free from this scourge for the space of eight years. This good fortune lasted for four years more, namely till the beginning of 1791, when it was doomed to suffer a terrible calamity. In the November preceding, a Ragusan vessel was freighted at Constantinople for the transport of a great number of Georgian, Circassian, and other young recruits for the service of the Beys of Egypt. In the course of the voyage, several of these had been carried off by plague, as well as the captain of the vessel, and some of the sailors. The vessel was obliged to stop at Rhodes to recruit her crew, and cast anchor at Alexandria in December 1790. As soon as this event was known, and also the circumstance, that the plague had been raging at Constantinople when the vessel sailed, a general apprehension prevailed in Egypt, that the contagion would explode in the spring following. Many of the Mamelukes died soon after their debarkation at Alexandria, and in their route to Cairo. Some accidents happened from time to time in particular houses, but only in those of the Beys. The Sheck El Beled had a box of furs consigned to him by the vessel above-mentioned, on opening which, two of the domestics were seized with the plague and quickly carried off. In the month of March, the predicted event took place. The plague burst forth with unexampled fury and fatality—first among the Beys, but soon afterwards from towns to villages, till it had over-run almost the whole of lower and upper Egypt! More than two villages were entirely depopulated, and in Cairo alone, the mortality was seldom less than three, and often as high as six thousand *per diem*, from March till June, 1791! At the conclusion of this tragic scene, the keys of 7000 desolated houses were consigned to the care of Ismael, Pacha of Cairo, who, more fortunate than El Beled, and almost all the Beys of Egypt, escaped this dreadful scourge.

To attribute this destructive malady to any deleterious miasmata in the air, is, in our author's opinion, most preposterous. The air of Egypt is proverbial for its clearness and general salubrity—and if the causes of the plague were endemic, why did they lie dormant for twelve years, and only shew themselves soon after the arrival of a vessel unequivocally bearing the disease among her crew? Let us now turn to the sanitary laws so much despised by the Medico-Philosophists of our times.

No sooner had this explosion of plague taken place at Cairo, and other places, than the European merchants at Alexandria hastened to close their accounts with the people of the country, and prepare for close segregation. The French merchants, with their consulate, secluded themselves in a pile of buildings having but one common entrance from without, yet with a free communication within by means of a common corridor and galleries. As soon as all had signed the agreement for seclusion, our author drew up a code of quarantine, or rather of sanitary laws, to the observance of which they all took a solemn oath. Two Commissaries, relieved every twenty-four hours, were appointed to see the laws carried into the most rigid execution—and nothing could be received or sent out without their express permission and supervision. Three barriers, within the common entrance, were established as additional checks and preventives against the introduction of suspected articles—and, in short, the most scrupulous measures were adopted to guard against contagion. The detail of these precautionary measures we may pass over; but we shall draw for a moment the attention of our readers to a circumstance which occurred at Cairo. The French merchants there segregated themselves as at Alexandria, but the buildings which they inhabited had each an entrance into the street, so that the observance of the sanitary laws was left at the mercy of each individual family. Among these was the Sieur Mélan who, one day on being apprised of the illness of a Bey who owed him a sum of money, broke the code of restrictions, and had communication, though in a very slight degree, with a few whom he employed. In a few hours afterwards he was seized with plague, and died, together with three of his servants!—But to return to the Alexandrian Franks. During the period above-mentioned (upwards of 120 days) this little garrison continued in the very focus of contagion—in the midst of thousands dead and dying of plague, yet perfectly free from that and all other diseases. "Nous nous trouvâmes au centre de la contagion, non-seulement spectateurs passifs de ses ravages mouïs, mais encore exempts de toutes maladies." As great mortality prevailed among the Frank seamen in the port of Alexandria, our author caused as many as possible of them to be brought under the walls of the building, where he and the Surgeon-Major of the Navy examined them minutely, though rigidly avoiding actual contact. In this way they had opportunities of making many observations on the symptoms of this disastrous scourge. Of all the phenomena, there were only two which were never absent—inflamed appearance of the eyes and countenance, and swelling of the tongue. These, together with a difficulty of speaking and a tottering gait, were certain criteria of the plague. The other symptoms were far from being uniform or constant. The pain of head and nausea by which some were tormented, did not obtain at all in others. Inflamed throat was more constant than most other phenomena, with the exception of the characteristic symptoms above-mentioned. There were instances where no eruption or buboes appeared; but, in general, there were from one to seven buboes exhibited in each case. Some presented petechiæ—and some an eruption resembling flea-bites. The pulse could not be examined, for obvious reasons. He who hazarded such an experiment, would not have lived many days to tell the tale! But whatever was the variety of symptoms, there was unfortunately only one mode of termination. *Not a single sailor, from March till June, recovered from this dreadful malady!* Death took place between the third and the seventh days. This is a melancholy portrait in a *therapeutical* point of view; but it is exceedingly interesting and important in its *prophylactic* relations. If we cannot *cure* a disease, it is surely a reason why we should use every precaution to *prevent* it.

Early in June, the atmosphere of Egypt began to present some clouds, which drifted incessantly, and with extreme rapidity, during three months in succession, from N.W. to S.E. to be lost among the mountains of Ethiopia, and to supply ultimately the waters of the Nile. These flying clouds diffused a vapoury moisture through the air, which penetrated into every thing—so that linen locked up in the securest manner, became quite damp in a short time. To this heavy dew the Egyptians are indebted for delivery from the plague. In the course of 15 or 20 days it invariably extinguishes every symptom and every germ of the disease. Even from the very commencement of these dews, the plague becomes greatly mitigated in its fatality.

Dr. Maclean and his disciples will tell us that the rise and cessation of plague at particular periods of the year, are proofs of its being a product of the soil or of the air—but they will not condescend to explain why small-pox, measles, and other diseases, acknowledged to be contagious, are greatly influenced and modified (as to their spread or development) by particular seasons and periods of the year. And how did this little secluded band of Franks manage to breathe the same air and live in the very midst of the dead and dying for four months, without suffering any inconvenience; whilst their less fortunate countrymen, exposed to communication with the inhabitants, almost all died? The conclusion which every unbiassed mind must draw from such a fact is irresistible; and yet the non-contagionist will not pay the slightest attention to it. Their business is not with *facts*—they hate the name of *facts*. All they want is paper on which to array *words*. Because we are not acquainted with *all* the laws which govern the causes of plague, it is attempted to renounce those laws which we do know. Because we cannot explain why a fall of rain or repeated falls of deavy dews in Egypt annihilate the germs of plague, we are also to disregard *seclusion*, which we do know to be a certain preventive of the malady! Because the plague is not every year in such a state of virulence at Constantinople as to cause its dissemination thence to the neighbouring countries, the non-contagionists would persuade us not to believe that it ever was or can be carried by men or goods through the Dardanelles! Because the port of Alexandria is constantly open to vessels from plague countries—because the climate of Egypt is, *in general*, so inimical to the existence of plague or its cause, as to annihilate both the moment they arrive there—because the inhabitants take no precautions of a quarantine nature; are we to infer that the contagion of plague is a chimera—that it never entered the port of Alexandria—that it never spread among the inhabitants—and that the seclusion of the Franks, was an unnecessary precaution against a phantom which had no existence?

Among the facts which our author has ascertained during his long residence in Egypt is, the complete protection which one attack of the plague in that country gives against future accidents. This circumstance he has verified by repeated observations.

But to Constantinople, where our author resided nine years, ardently endeavouring to investigate the nature and cause of this fatal scourge. In this ancient capital, where theorists have not been able to find lakes, marshes, or other sources of pestilential miasmata, the plague is, as it were, domiciliated, so as never to be entirely out of view. It is, therefore, looked upon as small-pox or measles, which must come sooner or later—in a milder or more aggravated form. This disease does not produce such destructive ravages in Constantinople as in most other countries, nor is it equally prevalent in all years. It has its phases of lenity and destructiveness, through which it runs its mysterious course—sometimes appearing highly contagious, and at others very little so. It mocks equally the timid and the courageous. The seasons and the elements appear to have no remarkable influence over it. When it is at its lowest ebb, the mortality is perhaps not more than triple the common or natural ratio of a large city like Constantinople; while, at its height, there are daily immolated at its shrine from four to five hundred individuals. When it happens to continue a few years unusually mild, there is generally what is called a *restitution*, when (as in the year after the great Egyptian plague) fifteen hundred corpses have been counted issuing in one day through the single gate of Adrianople! In general, however, it is milder at Constantinople than in most other countries—its course being slower—and the proportion of recoveries to deaths greater. But the most remarkable singularity of the Byzantine plague, (and which, if true, as it is positively asserted to be by our author, must completely defy human sagacity to unravel) is, that it is almost incapable of affecting any but the inhabitants of the Levant. This is a fact, our author avers, which he has verified by nine years attentive observation. The exceptions are so very rare as not at all to affect the truth of the general rule. And hence it is, that the Franks take no precautions against the plague. "I have seen," says M. De Butel, "the plague ravaging the quarters of Galata and Pera, whilst the European Ambassadors were giving a succession of fêtes, balls, and assemblies, consisting of several hundred persons in the interior of their residences, while at least an equal number of lacqueys were lounging in the anti-chambers—and this at a time when the plague was ravaging the habitations of the Turks and Greeks in every direction around them—all this too without producing the least alarm in the minds of the Europeans, who considered themselves, and were in reality, perfectly secure from the malady that surrounded them." Hence it is, that we see strangers from all parts of the world perambulating the streets, strolling through the bazaars, and frequenting the public places of Constantinople, without becoming infected with the plague. A curious circumstance may here be mentioned, namely that, one year out of the nine, which our author passed at Constantinople, was signalized by a total cessation of the plague; but at this period the air became so corrupted that a fever of a very malignant nature spread epidemically, and proved exceedingly destructive to the Franks, who lost more in that one season than they had done during the whole of the nine years which our author passed in that capital. M. Butel is of opinion, and we agree with him, that, in modern times, many fevers have been confounded with the true plague, and that, thus, much discrepancy of opinion has crept into the discussions of medical men. He has come to the conclusion, certainly from an experience and sphere of observation unequalled in the present day, "1^mo.^ *That the plague is communicable only by contact—that its miasms attach themselves to every material capable of harbouring them, and have no chemical affinity for atmospheric air, which is incapable of sustaining or transporting any particle of these miasms—that, consequently, a person may enter the room in which a patient with plague is confined, with perfect safety, as long as personal contact is avoided.* 2^ndo.^ *That those countries which are infested with malaria, marsh exhalations, and other causes of fever, not only do not engender plague (non-seulement n'engendrent point la peste) but are no more exposed to its ravages than the most healthy countries and cities.*" Speaking of the plague in Egypt, our author observed that the houses of the Beys, which were unquestionably the cleanest and most airy in the country, were the first to be infected, and, in short, completely depopulated. To this observation, which is of the greatest importance, he adds that, he has several times seen the plague at Constantinople ravage those quarters of the city which were the most elevated, and consequently enjoying the purest air, and inhabited by the better classes of society, while the quarter of Balata, the lowest in the capital, at the bottom of the harbour, a kind of Cloaca inhabited by Jews (au fond du port, espece de cloaque habité par les Juifs) the very dregs of the people, was completely exempt from the disease. 3^tio.^ That no remedy has hitherto been discovered of any avail against the true plague—and, consequently, that all recoveries are owing to Nature; or in other words, to the efforts of the constitution.

It was remarked, that there was something in the nature of the plague at Constantinople, or of the European constitution, which rendered the latter very unsusceptible of the contagion. Granting that this be the case, for which, however, we can by no means vouch, there can be no doubt that, as the disease appeared in Egypt, it was highly contagious, as our military officers, as well as those of the French army, had dear-bought reason to know. It is evident too that, so late as 1813, it produced dreadful devastation in Malta, whatever was the source whence it came. It is also known that Dr. Maclean caught the plague while in the Greek Pest-house in Pera—and our author records the case of an English physician, who inoculated himself (we suppose he alludes to Dr. Whyte) with matter from a pestilential bubo, and took the disease, though he did not *then* die of it, but six months afterwards, as taken in the natural way. All considerations, therefore, induce us to agree with the concluding sentiment of our author. "Amidst the mysteries and obscurities which encompass the subject of plague, one truth is clear, the existence of certain means of prevention or preservation, which Providence has placed in our hands. It is the only antidote against this dreadful poison. This is QUARANTINE." It would be well for our senators to pause on such documents as these, and consider well, before they abrogate those sanitary laws, which we have strong reason to believe, are the surest bulwark against the introduction of a disease over which medicine has so little control.

### ALLEDGED INFANTICIDE.

A married woman was indicted at the Sussex Assizes, August, 1825, for the murder of her infant, under the following circumstances. She was servant in a family at Worthing, and dismissed when found to be pregnant. She took lodgings, but was delivered of a child in a field near Bellingshurst on the 14th May, 1825. She returned to her lodgings, and her appearance exciting the suspicions of her landlady, she first denied, but afterwards acknowledged that she was delivered of a child, while getting over a style—that the child in falling to the ground was killed on the spot—and that she had buried it in a ditch. The child was found in a muddy ditch, its head fractured, and some of the mud in the trachea, bronchia, and even as far as the air-cells of the lungs. The surgeons examined, gave it as their opinion, that the child's death might have been occasioned in the manner stated by the mother, and that, supposing the child to be apparently dead from the fall, it might have sufficient life to imbibe, by convulsive respiration, the liquid mud found in the windpipe and lungs. The prisoner was acquitted.

Mr. Martin, surgeon, of Pulborough, who has published this case, has also appended some remarks, and stated some experiments bearing on the point. A rabbit was drowned in a trough filled with dirty water. In twenty minutes afterwards it was examined, and the bronchia were found filled with dirt and froth—the air-cells were found injected with dirt. Several other experiments, all coming to nearly the same result, were made; but one of them deserves particular notice. "A cat was dashed against a wall and placed in the trough, in a state of insensibility, and died with so little struggle, that it seemed to make no effort to breathe. The lungs were nearly in the same state as the rabbit's in the last experiment—a little dirt had found its way into the bronchia and some of their larger branches."

We acknowledge that appearances were exceedingly strong against this woman; but still as there was a *possibility* that the circumstances were such as she stated, we think the Jury gave a proper verdict. In the experiment last stated, the mud had found its way into the bronchia, although the cat might be considered as killed before immersion. Now if the child had dropped suddenly from the mother, as we know it sometimes does, and, if in staggering she had trampled on the head of the fœtus, the fractures mentioned above might have been produced. In the state of mind in which this unhappy woman was, she might have buried the child, from the very fear of being suspected of infanticide, after such an accident to the head. And as there must necessarily have been much precipitation in such a transaction, some small remains of life might have still existed in the infant, and some convulsive motion of the diaphragm and other muscles might have produced the inhalation of the muddy water. We confess that this line of argument is pushing mercy to its utmost stretch of clemency—perhaps of credulity—but if there be any case in which mercy should predominate over justice, it is in cases of infanticide. In this, as in suicide, we believe that the *mind* of the actor is deranged nine times out of ten. The suicide suffers the greatest punishment which human law can inflict—death. The infanticide must suffer more than the pains of death, in working herself up to such a violation of the strongest of Nature's ties. If then there be the smallest *possibility* of innocence, or of momentary insanity, let her have the benefit of it. The tone of Mr. Martin's remarks seems more inclined towards rigid justice, in such cases, than those which we have ventured to make; but as God is supposed to be merciful as well as just, why should not man imitate his Creator?

